# Toward Single-Time-Point Image-Based Dosimetry of ^177^Lu-PSMA-617 Therapy

**DOI:** 10.2967/jnumed.122.264594

**Published:** 2023-05

**Authors:** Julia Brosch-Lenz, Astrid Delker, Friederike Völter, Lena M. Unterrainer, Lena Kaiser, Peter Bartenstein, Sibylle Ziegler, Arman Rahmim, Carlos Uribe, Guido Böning

**Affiliations:** 1 Department of Nuclear Medicine, University Hospital, LMU Munich, Munich, Germany;; 2 Department of Integrative Oncology, BC Cancer Research Institute, Vancouver, British Columbia, Canada;; 3 Department of Radiology, University of British Columbia, Vancouver, British Columbia, Canada;; 4 Department of Physics, University of British Columbia, Vancouver, British Columbia, Canada; and; 5 Department of Functional Imaging, BC Cancer, Vancouver, British Columbia, Canada

**Keywords:** single-time-point dosimetry, ^177^Lu, PSMA therapy

## Abstract

Radiopharmaceutical therapies (RPTs) with ^177^Lu-prostate-specific membrane antigen (PSMA) ligands have demonstrated promising results for the treatment of metastatic castration-resistant prostate cancer. The lack of absorbed-dose–effect relationships currently prevents patient-specific activity personalization. To ease the implementation of dosimetry in the routine clinical workflow for RPT, simplified methods such as single-time-point (STP) instead of multiple-time-point (MTP) imaging protocols are required. This work aimed at assessing differences in the time-integrated activity (TIA) of STP versus MTP image-based dosimetry for ^177^Lu-PSMA-617 therapy. **Methods:** Twenty metastatic castration-resistant prostate cancer patients with MTP quantitative ^177^Lu-SPECT imaging data (∼24, 48, and 72 h post injection (p.i.)) available on first and second ^177^Lu-PSMA-617 therapy cycles were included in this study. Time–activity curves were fitted for kidneys and lesions to derive effective half-lives and yield a reference TIA. STP approaches involved the formula by Hänscheid (STP_H_) and a prior-information method (STP_prior_) that uses the effective half-lives from the first therapy cycle. All time points were considered for the STP approaches. Percentage differences (PDs) in TIA between STP and MTP were compared for the second therapy cycle. **Results:** Using STP_H_ at 48 h p.i. for kidneys showed a −1.3% ± 5.6% PD from MTP, whereas STP_prior_ showed a PD of 4.6% ± 6.2%. The smallest average PDs for the 56 investigated individual lesions were found using STP_prior_ at 48 h p.i., at only 0.4% ± 14.9%, whereas STP_H_ at 72 h p.i. had a smallest PD of −1.9% ± 14.8%. **Conclusion:** STP dosimetry for ^177^Lu-PSMA-617 therapy using a single SPECT/CT scan at 48 or 72 h p.i. is feasible, with a PD of less than ±20% compared with MTP. The validity of both STP_H_ and STP_prior_ has been demonstrated. We believe this finding can increase the adoption of dosimetry and facilitate implementation in routine clinical RPT workflows. Doing so will ultimately enable the finding of dose–effect relationships based on fixed therapy activities that may, in future, allow for absorbed-dose–based RPT activity personalization.

## Visual Abstract

**Figure:**
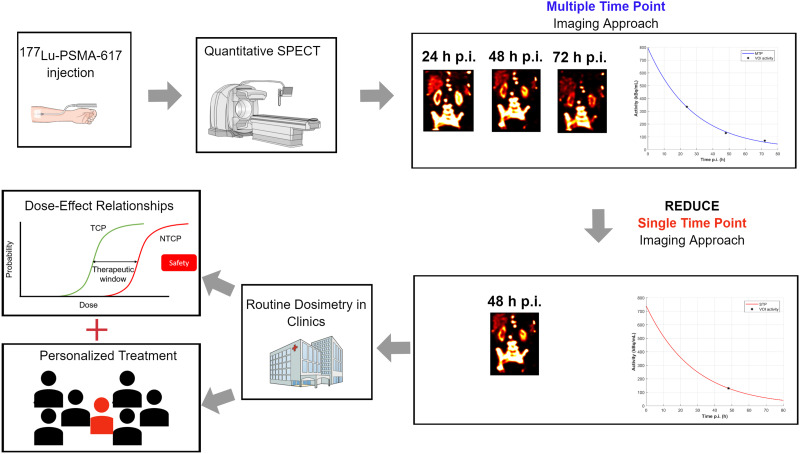

Radiopharmaceutical therapy (RPT) targeting the prostate-specific membrane antigen (PSMA) has shown significant promise in the treatment of metastatic castration-resistant prostate cancer (mCRPC) (1–3). PSMA radioligand therapy with ^177^Lu was first conducted in 2013 (4), and shortly afterward, dosimetry results were reported for ^177^Lu-PSMA-617 (5). Considerable improvements in overall survival and radiographic progression-free survival for mCRPC patients receiving ^177^Lu-PSMA-617 therapy plus the standard of care, against the standard of care alone in the VISION trial (NCT03511664) (1), led to approval by the U.S. Food and Drug Administration in 2022. Although some evidence of the advantage of dosimetry-based treatment personalization has been shown recently for ^90^Y liver radioembolization (6), current practice for most RPTs relies on fixed injected activities. The therapeutic scheme for ^177^Lu-PSMA therapy involves 4–6 therapy cycles with fixed activities (7), whereas optimal patient treatment would consider individual factors during RPT planning, such as weight, height, tumor burden, pretreatments, dosimetry, and patients’ preferences (8). The lack of broadly available absorbed doses (ADs) for RPT prevents reliable dose–effect relationships for lesions and healthy organs from being obtained, impeding treatment personalization in terms of activity and number of cycles (9). The possibility of correlating pretherapy information with dosimetry and patient outcome was recently shown (10) and should motivate the community to implement routine dosimetry within RPTs and actively plan and adapt an RPT to personalize treatment and maximize patient therapeutic benefit.

The evidence of patient benefit from personalized RPTs is limited by the fact that image-based dosimetry is still not routinely implemented along with RPTs. One limitation preventing clinical adoption of individualized dosimetry is that pharmacokinetic measurements typically require image acquisitions at multiple time points (MTPs) post injection (p.i.) of the radiopharmaceutical. Other factors, such as limited clinical resources (e.g., scanner availability and personnel), as well as the additional costs of MTP imaging and the unclear reimbursement (11), limit the application of personalized dose assessments. This lack of clinical adoption, however, goes against European council directive 2013/79/Euratom, which requests individual planning and verification of exposed target volumes and minimization of dose to nontarget regions, according to the ALARA principle (12).

In this work, we aimed to assess single-time-point (STP) image-based dosimetry for ^177^Lu-PSMA-617 therapy for the second therapy cycle. Specifically, we considered the formula by Hänscheid et al. (STP_H_) (13) and a prior-information approach (STP_prior_) that uses MTP imaging during the first therapy cycle and STP imaging for subsequent cycles. We believe that validation of a simple dosimetry approach that requires a single SPECT/CT scan can increase the adoption of dosimetry and facilitate implementation in routine clinical RPT workflows. Doing so can enable the finding of dose–response relationships based on fixed therapy activities that will ultimately allow for AD-based RPT activity personalization.

## MATERIALS AND METHODS

### Patients

This study was conducted on a cohort of patients with mCRPC who received two 6-GBq cycles of ^177^Lu-PSMA-617. Twenty patients with MTP imaging data available for both therapy cycles were included. Therapeutic injections and subsequent imaging were performed at the department of nuclear medicine of the university hospital of Ludwig Maximilian University of Munich. Data were irreversibly anonymized. The institutional ethics committee approved this retrospective study (approval 21-0618), and the requirement to obtain informed consent was waived.

### Imaging Protocol

**FIGURE 1.**
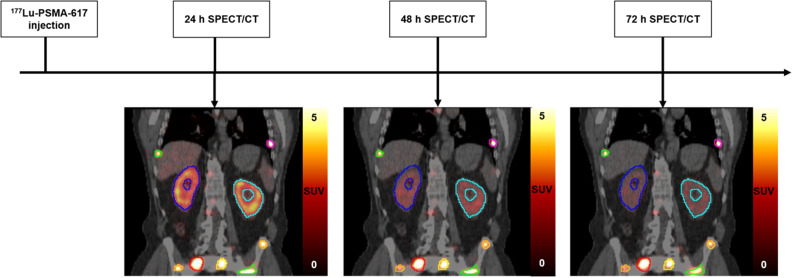
Overview of MTP imaging protocol.

The details of the MTP imaging protocol (Fig. 1) are in the supplemental materials (available at http://jnm.snmjournals.org) (5*,*^14^–^17^).

### Determination of Time–Activity Curves

Images were processed using PMOD (version 4.005; PMOD Technologies LLC). The 24 h p.i. SPECT scan of each therapy cycle was chosen as a reference image to which the 48 h p.i. and 72 h p.i. SPECT scans were rigidly registered. Segmentation was performed on the 24 h p.i. SPECT scans of each cycle. The kidneys were segmented by applying a 20% fixed threshold, which produced good alignment when overlying the kidney volumes of interest (VOIs) on the CT scan, excluding the kidney pelvis. Manual adjustments were made when necessary. The qPSMA approach of Gafita et al. (18) was adopted for segmentation of individual lesions on the 24 h p.i. SPECT scan per cycle, which was converted into standardized uptake values (SUVs) based on body weight. The determined patient- and cycle-specific threshold was applied to the 24 h p.i. SPECT scan with an automatic multiregion approach. Physiologic uptake regions that were mistakenly selected as VOIs by the automatic multiregion threshold approach, such as in the gastrointestinal tract or bladder, were removed. Lastly, a whole–field-of-view (FOV) tumor burden (TB_FOV_) VOI containing all individual lesions was created. The lesion segmentation was verified and, if necessary, manually adjusted on the SPECT and CT scans by 2 experienced readers in a consensus reading.

All VOIs were copied to the coregistered 48 h p.i. and 72 h p.i. SPECT scans, and the activity values of each VOI were extracted to generate time–activity curves. These were fit to a monoexponential function using MATLAB (version R2019b; The MathWorks, Inc.) to determine the effective half-lives (T_1/2 eff_) (17) for kidneys, TB_FOV_, and individual lesions. The procedure was performed for both therapy cycles.

### Time-Integrated Activity (TIA) with MTP and STP Approaches

The TIA for each VOI in the second therapy cycle was calculated using 3 different methods: the first used the monoexponential fit with all points available from the MTP scans in the second cycle (considered the reference TIA (TIA_ref_), determined from activity at time *t* = 0 for the second therapy cycle, $A_{0}^{2\text{nd}}$, and T_1/2 eff_ for the second therapy cycle, ${\text{T}}_{\text{1/2}\;\text{eff}}^{\text{2nd}}$ [Eq. 1]); the second used T_1/2 eff_ determined from the curve fitting of the first cycle (${\text{T}}_{\text{1/2}\;\text{eff}}^{\text{1st}}$, prior information) and the STP activity value of the second cycle; and the third used the approach suggested by Hänscheid (13).

TIAref=A02ndln2T1/2 eff2nd Eq. 1

Three different STP TIAs were calculated for the second method, STP_prior_, with Equation 2 by combining ${\text{T}}_{\text{1/2}\;\text{eff}\;}^{\text{1st}}$ with the single activities *A*(*t*) measured at time *t* = 24, 48, or 72 h p.i.

$${\text{STP}}_{\text{prior}}\;\text{TIA}=\frac{A\left(t\right)\cdot 2^{\frac{t}{{\text{T}}_{\text{1/2}\;\text{eff}}^{\text{1st}}}}}{\frac{\text{ln}2}{{\text{T}}_{\text{1/2}\;\text{eff}}^{\text{1st}}}}$$ Eq. 2

The third method, STP_H_, estimated the STP TIA using the method of Hänscheid (13). This approach assumes that if the imaging time point *t* is within the interval from 0.75 to 2.5 times the T_1/2 eff_ of the respective VOI, one can replace Equation 2 by a simplified formula (Eq. 3) with less than 10% error in TIA compared with MTP. Three different STP TIAs were calculated using the activities *A*(*t*) measured at time *t* = 24, 48, or 72 h p.i.

$${\text{STP}}_{\text{H}}\;\text{TIA}\approx \frac{A\left(t\right)\cdot 2\cdot t}{\text{ln}2}$$ Eq. 3

### Comparisons

The STP approaches for the second therapy cycles were compared with the MTP reference. The percentage difference (PD) in STP TIA versus TIA_ref_ was calculated for each kidney, for TB_FOV_, and for up to 6 lesions per patient if they were visible in the FOV of both cycles. Bland–Altman plots were used to compare the STP approaches with MTP (19*,*^20^).

### Statistical Analyses

Statistical analysis used the Wilcoxon signed-rank test for comparisons between MTP and each STP approach and between the T_1/2 eff_ of the first and second cycles.

## RESULTS

Unless otherwise stated, all reported values are given as average ± SD (minimum; maximum).

### Patients

Twenty patients with mCRPC were included in this analysis. The average administered activity of ^177^Lu-PSMA-617 for all patients and therapy cycles was 6.09 ± 0.13 GBq (5.74; 6.70 GBq). Left and right kidneys were analyzed separately. The patients’ TB_FOV_ volume averaged 462 ± 361 ml (8; 1,229 ml). One patient had no lesions within the SPECT FOV. In total, 56 lesions that were seen within the FOV for the first and second therapy cycles were analyzed.

### Distribution of Effective Half-Lives

**FIGURE 2.**
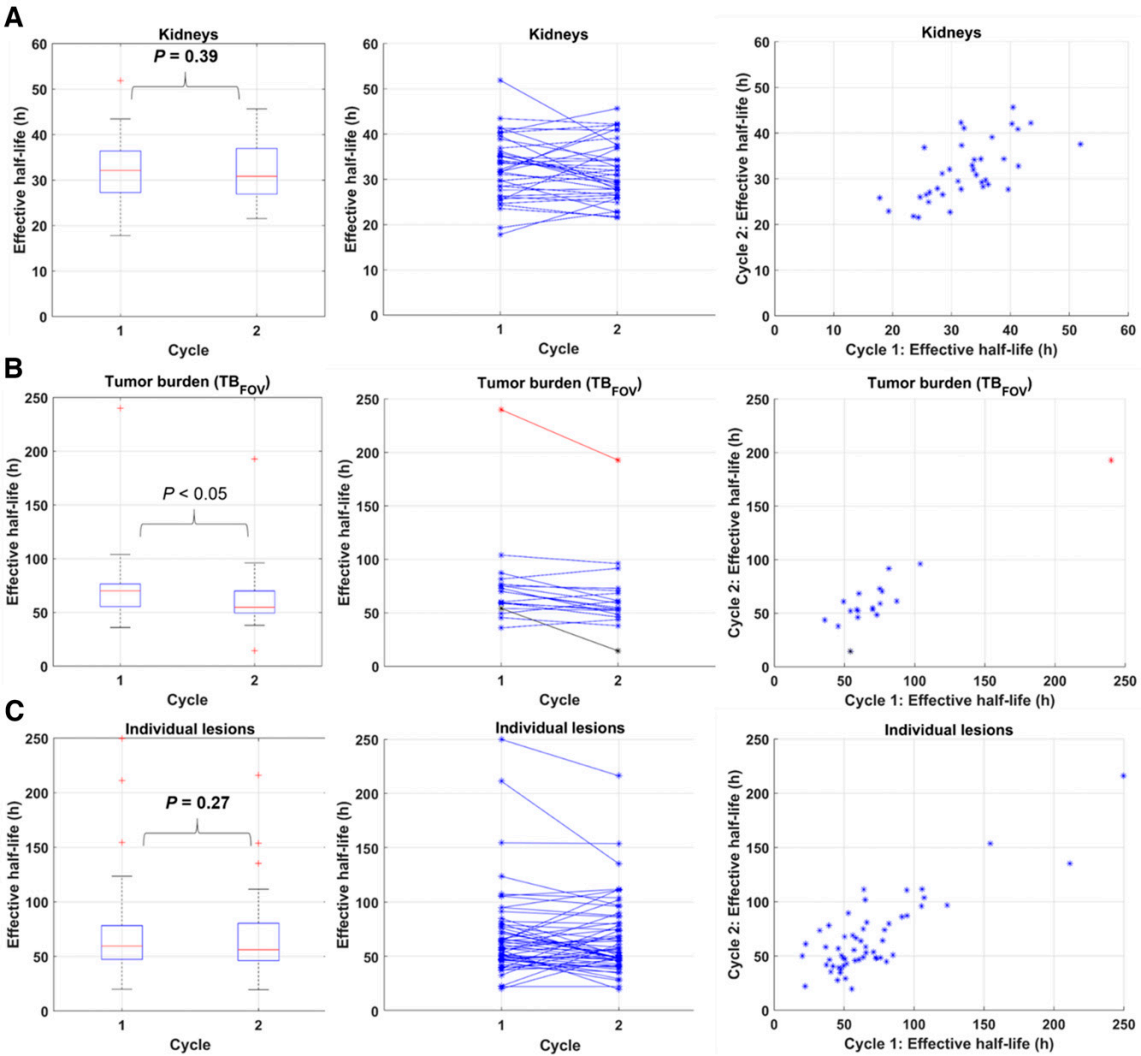
Distribution of T_1/2 eff_ calculated using MTP method for kidneys (A), TB_FOV_ (B), and individual lesions (C) for both therapy cycles. Plots further include results of statistical analysis using Wilcoxon signed-rank test for T_1/2 eff_ between cycles 1 and 2.

Figure 2 shows the T_1/2 eff_ distributions obtained with the MTP approach. The average T_1/2 eff_ for the first and second therapy cycles was 32.5 ± 7.0 h (17.8; 51.9 h) and 31.7 ± 6.4 h (21.6; 45.7 h), respectively, for kidneys; 75.3 ± 41.8 h (45.5; 240.0 h) and 64.8 ± 35.0 h (14.5; 192.8 h), respectively, for TB_FOV_; and 69.0 ± 40.0 h (20.1; 249.7 h) and 66.6 ± 34.2 h (19.7; 216.2 h), respectively, for individual lesions. Twenty-six of the 56 investigated lesions had a T_1/2 eff_ PD of more than ±20%.

When T_1/2 eff_ obtained with the MTP approach was compared between the first and second therapy cycles using the Wilcoxon signed-rank test, significant differences (i.e., *P* < 0.05) were found for TB_FOV_ (*P* = 0.02) (*n* = 19; 1 patient had no lesions) but not for kidneys (*P* = 0.39) (*n* = 37; 3 patients had only 1 active kidney) or individual lesions (*P* = 0.27) (*n* = 56).

### Comparison of TIA with Respect to STP Approaches

**FIGURE 3.**
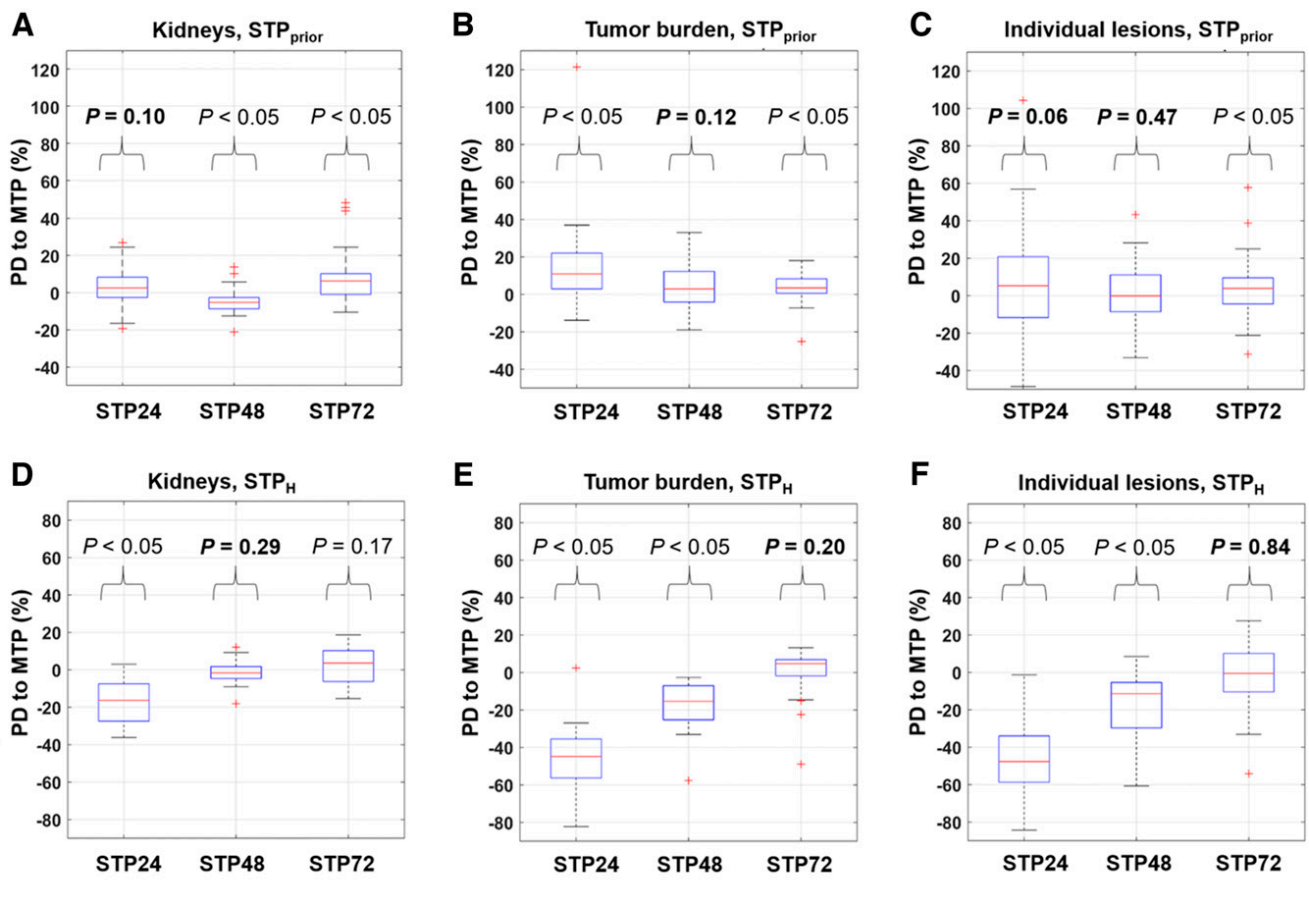
Distribution of PD of TIA in STP_prior_ (A–C) and STP_H_ (D–F) vs. MTP reference for kidneys (A and D), TB_FOV_ (B and E), and individual lesions (C and F). Plots further include results of statistical analysis using Wilcoxon signed-rank test between MTP and each respective STP approach.

Figure 3 shows the PDs in TIA between the MTP and STP approaches. Supplemental Table 1 displays the values.

**FIGURE 4.**
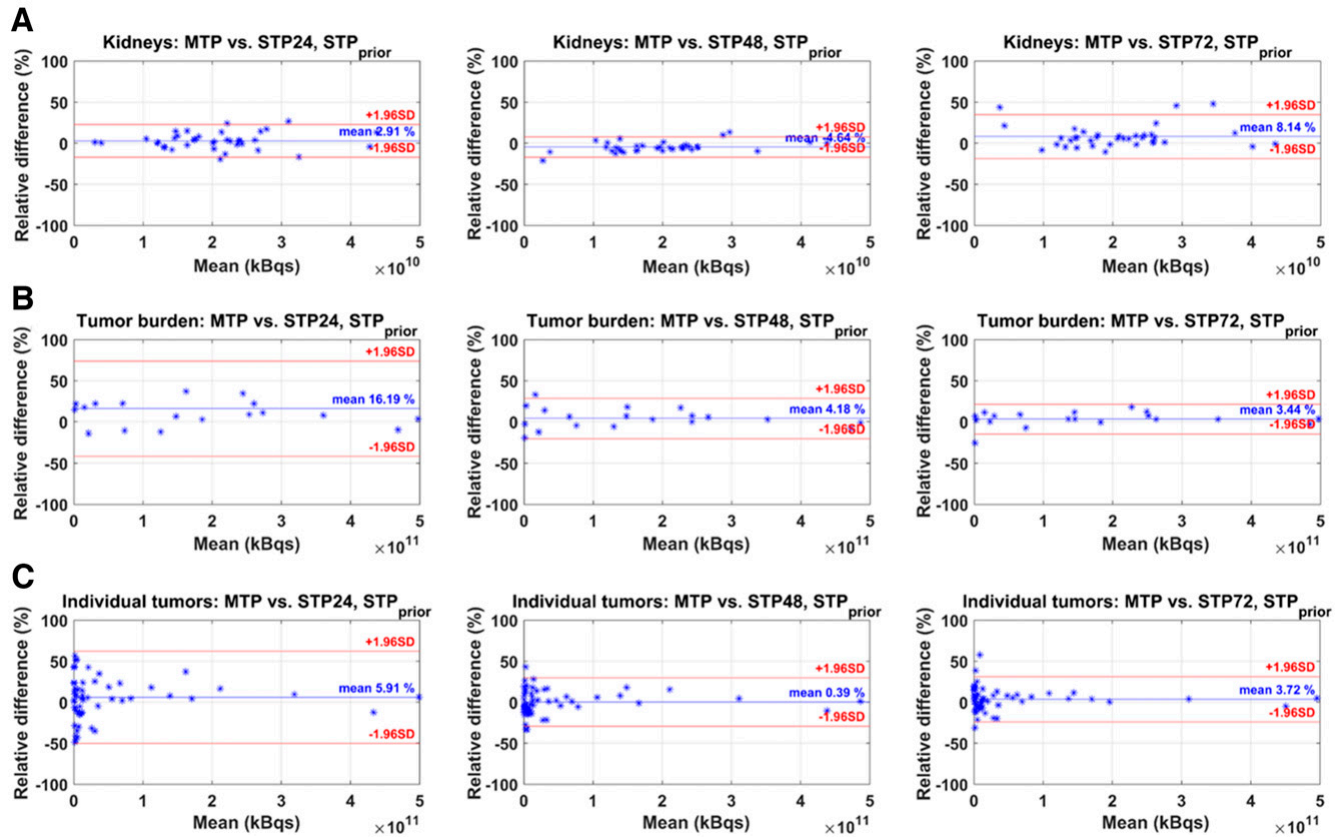
Bland–Altman plots of STP_prior_ vs. MTP reference for kidneys (A), TB_FOV_ (B), and individual lesions (C). STP24 = STP at 24 h p.i.; STP48 = STP at 48 h p.i.; STP72 = STP at 72 h p.i.

**FIGURE 5.**
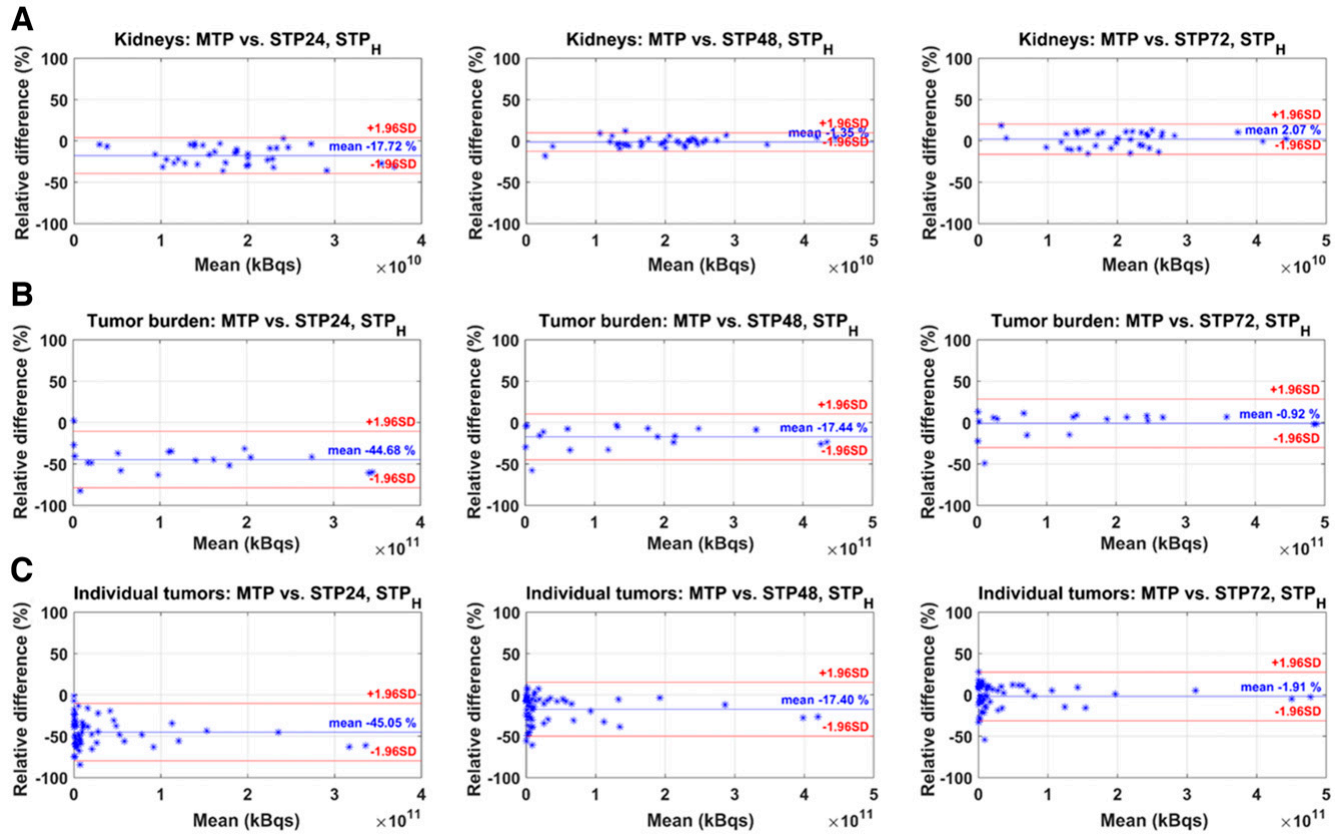
Bland–Altman plots of STP_H_ vs. MTP reference for kidneys (A), TB_FOV_ (B), and individual lesions (C). STP24 = STP at 24 h p.i.; STP48 = STP at 48 h p.i.; STP72 = STP at 72 h p.i.

The Bland–Altman plots of STP_prior_ and STP_H_ compared with MTP are given in Figures 4 and 5. The mean relative difference between MTP and STP_prior_ was closest to zero for kidneys at 24 h p.i., for TB_FOV_ at 72 h p.i., and for individual lesions at 48 h p.i. (Fig. 4). However, the limits of agreement were smallest for kidneys at 48 h p.i., for TB_FOV_ at 72 h p.i., and for individual lesions at 48 h p.i. For STP_H_, the difference from MTP was closest to zero, with the smallest limits of agreement at 48 h p.i. for kidneys and at 72 h p.i. for individual lesions (Fig. 5). For TB_FOV_, the difference was smallest at 72 h p.i., whereas the limits of agreements were slightly smaller at 48 h p.i.

### Statistical Analyses

The results of the statistical analysis for the STP approaches compared with the MTP reference are shown in Figure 3. In general, no significant difference in TIA for kidneys was found for an STP_prior_ at 24 h p.i. or an STP_H_ at 48 h p.i. For TB_FOV_, no significant difference in TIA was found for an STP_prior_ at 48 h p.i. or STP_H_ at 72 h p.i. Lastly, for individual lesions, no significant difference in TIA was found for an STP_prior_ at 24 h p.i., STP_prior_ at 48 h p.i., or STP_H_ at 72 h p.i.

Table 1 summarizes the number and percentage of VOIs for which the imaging time points per therapy cycle were within the interval from 0.75 to 2.5 times the T_1/2 eff_ of that region as calculated with the MTP approach. The imaging time point at 48 h p.i. lay within that range for 97% and 100% of kidneys for both cycles 1 and 2, whereas for TB_FOV_ and individual lesions, the largest number of VOIs within that range was at 72 h p.i. However, for 25% of individual lesions and 21% of the TB_FOV_ VOIs, 72 h p.i. was outside the interval for cycle 2.

**TABLE 1.** Number of VOIs for Which Imaging Time Point was Within Interval from 0.75 to 2.5 Times T_1/2 eff_ of Cycle 1 or 2

Parameter	Cycle	VOIs (*n*)		
		24 h p.i.	48 h p.i.	72 h p.i.
Kidneys (*N* = 37)	1	7 (19%)	36 (97%)	28 (76%)
	2	12 (32%)	37 (100%)	27 (73%)
TB_FOV_ (*N* = 19)	1	0 (0%)	6 (32%)	17 (89%)
	2	1 (5%)	9 (47%)	15 (79%)
Individual lesions (*N* = 56)	1	3 (5%)	26 (46%)	43 (77%)
	2	2 (4%)	30 (54%)	42 (75%)

**FIGURE 6.**
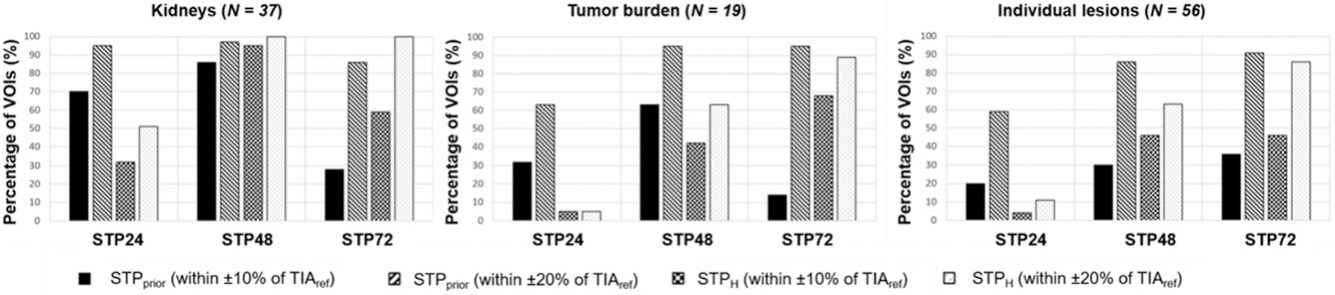
Percentage of VOIs for which difference in TIA for STP vs. MTP fell within ±10% or ±20%.

Figure 6 shows the percentage of VOIs for which the STP TIA was within ±10% and ±20% of TIA_ref_ for both the STP_prior_ and STP_H_ approaches. For STP_H_, 95% of kidneys were within ±10% of TIA_ref_ at 48 h p.i., compared with 86% for STP_prior_. For TB_FOV_, 95% of VOIs were within ±20% of TIA_ref_ at 48 h p.i. and 72 h p.i. for STP_prior_, compared with 68% and 89% for STP_H_, respectively. For STP_prior_, 86% and 91% of the individual lesions were within ±20% of TIA_ref_ at 48 h p.i. and 72 h p.i., compared with 63% and 86% for STP_H_, respectively.

## DISCUSSION

In this work, we aimed at comparing STP with MTP image-based dosimetry methods, which could increase clinical adoption. STP dosimetry methods have been studied predominantly for ^177^Lu-DOTATATE therapy (13*,*^21^–^23^) but also for ^177^Lu-PSMA therapy (24–26). Three different approaches for STP dosimetry have been proposed: population-based mean T_1/2 eff_ (27), using prior information from the first therapy cycle for subsequent cycles (26), and using the formula by Hänscheid et al. (13). The first approach has been suggested to be valid for calculation of kidney ADs in ^177^Lu-DOTATATE and ^90^Y-DOTATOC therapies (22*,*^27^). Given the mean T_1/2 eff_ of 32.5 ± 7.0 h p.i. and 31.7 ± 6.4 h p.i. for the first and second ^177^Lu-PSMA-617 therapy cycles determined from MTP imaging in this work, this approach may be a valid assumption. However, given the high variation and large spread of T_1/2 eff_ for TB_FOV_ and individual lesions (Figs. 2B and 2C), the population-based approach may not be suitable for lesion AD calculations in ^177^Lu-PSMA therapies. Therefore, we compared clinically feasible dosimetry approaches for kidneys and lesions with a reduced number of imaging time points based on STP_prior_ and STP_H_.

STP-based approaches showed smaller differences between TIA and TIA_ref_ for kidneys than for lesions. These differences can be associated with the smaller variations in T_1/2 eff_ (Fig. 2). For the STP_prior_ approach, our analysis indicated that an STP at 24 h p.i. results in TIA differences from MTP that are on average closer to zero (Fig. 3A). However, 48 h p.i. is more favorable if a smaller range of variations in PD versus TIA_ref_ is preferred (Figs. 3A and 4A). Our results agree with those reported by Kurth et al. (26), who applied the STP_prior_ approach for cycles 2–6 and found differences in AD of ±6% for kidneys and ±10% for parotid glands when using a single SPECT scan at 48 h p.i. of ^177^Lu-PSMA-617, compared with MTP. Our analysis also suggests that when using the STP_H_ approach, an STP at either 48 h p.i. or 72 h p.i. is favorable. However, an STP_H_ at 48 h p.i. may be optimal for kidney AD calculations given the smaller range of variations in STP TIA versus TIA_ref_ (Figs. 3B and Fig. 5A). For kidneys, STP_H_ outperformed STP_prior_ at 48 h p.i. in terms of PD in TIA with respect to MTP (Fig. 6). With STP_H,_ most (95%) kidney TIAs are expected to be within 10% of those calculated with MTP, with few (5%) falling within 10%–20%. For all kidneys except one, the 48 h p.i. imaging time point was within the interval from 0.75 to 2.5 times the T_1/2 eff_. STP_H_ therefore yielded TIA estimates very close to TIA_ref_. STP_prior_, on the other hand, relies on a comparable T_1/2 eff_ for cycles 1 and 2. We observed up to a 45% difference in T_1/2 eff_ for some investigated kidneys. However, this translated to a PD in TIA_ref_ of between only −6% and 14%, which could be tolerated as long as the overall kidney function of the patient was good before therapy and the cumulative kidney AD was far below the considered toxicity threshold of 23 Gy.

For TB_FOV_ and individual lesions, an imaging time point of 72 h p.i. seems optimal, as the ranges of PD when compared with MTP are the smallest (Figs. 3A, 4B, and 4C) for the STP_prior_ approach. Similarly for STP_H_, the PD when compared with MTP was closer to zero at 72 h p.i. (Figs. 3B, 5B, and 5C). However, to obtain TIA estimates for both kidneys and lesions in a single scan, an STP at 48 h p.i. might be a valid compromise. But this compromise comes at a higher variation in PD with respect to MTP for lesions.

STP_prior_ performed better overall for TB_FOV_ and individual lesions than did STP_H_ (Fig. 6). The performance of STP_H_ improved with later imaging time points. This finding agrees with findings reported by Hänscheid et al. for ^177^Lu-DOTATATE (13) and Jackson et al. for ^177^Lu-PSMA-617 (25), both of whom found better agreement between STP and MTP for lesions at imaging time points even beyond 72 h p.i. STP_H_ showed an overall underestimation of TIA for TB_FOV_ and individual lesions (Fig. 3B). A similar negative skew for STP_H_ was previously observed by Gustafsson and Taprogge (28), underlining that STP approaches are limited in accuracy and that the distribution of T_1/2 eff_ in a population must be carefully determined. Our results, however, suggest that STP_prior_ is more suitable for tumor dosimetry, especially if the time point is 48 h p.i., matching our recommendation for kidneys. For STP_prior_, it is expected that most TIAs will fall within 20% of those calculated with MTP. Our suggestion of performing SPECT at 48 h p.i. agrees with the analysis of Hou et al. (24). Generally, this recommendation is limited for STP_H_, since, as shown in Table 1, the imaging time point of 48 h p.i. was outside the interval from 0.75 to 2.5 times the T_1/2 eff_ for about 50% of the individual lesions for cycles 1 and 2 and for 50%–60% of TB_FOV_.

The hybrid MTP/STP (STP_prior_) approach presented here allows for collection of all required SPECT images during the routine 3-d hospital stay for patients receiving ^177^Lu-PSMA-617 therapy at our institution. This data collection should, however, still be feasible for other institutions with in-patient therapies and for centers that discharge patients on day 0 if they agree to return during the following 2 days. We understand that the latter situation is not optimal, but open communication with the patient highlighting the benefit of MTP imaging during first therapy cycle may increase the patient’s willingness to cooperate and participate in multiple scans. When a patient can tolerate only STP imaging (e.g., because of pain) or when only a single scan is feasible due to scanner availability or there are reimbursement issues, the STP_H_ approach can still be valid. However, imaging should be performed at 72 h p.i. or later (Fig. 6), when differences in TIA were within ±20% for all kidneys and for over 85% of the investigated TB_FOV_ and individual lesions. In our investigation, this imaging time point was within the interval from 0.75 to 2.5 times the T_1/2 eff_ for over 70% of kidneys, TB_FOV_, and individual lesions, as shown in Table 1.

Specific patient situations should be considered when STP methods are applied. The STP_prior_ approach may be more prone to deviations from TIA_ref_ for lesions in cases of progressive disease or fast response (Supplemental Fig. 1). Protection of healthy organs from radiation-induced toxicities trumps achieving the highest possible lesion doses. When considering the minimum and maximum PDs of −21% and 14% for kidney TIA achieved with an STP_prior_ at 48 h p.i., and of −18.1% to 12.1% with STP_H_, these PDs bear the risk of under- or overestimation of the actual kidney dose. Dose underestimation in the individual patient may lead to application of subsequent therapy cycles even if the kidney dose threshold has already been exceeded. ADs obtained from STP methods should therefore be interpreted with caution, in view of the approximately 20% underestimation in a few patients. The condition and kidney function of the individual patient before and during treatment must be closely monitored to prevent radiation-induced toxicity. Our analysis revealed large minimum and maximum PDs of −19% to 33% for TB_FOV_ and −33% to 43% for individual lesions for an STP_prior_ at 48 h p.i., and of −58% to −3% for TB_FOV_ and −61% to 8% for individual lesions when using STP_H_. Since current clinical practice focuses on protection of healthy organs, these large ranges will likely not influence the patient’s course of treatment. However, this variation in lesion AD, with possible over- or underestimation of the actual lesion AD, can potentially impact the derivation of dose–response relationships for prostate cancer lesions. The research community should therefore focus on MTP-derived lesion ADs to determine the response of lesions to ^177^Lu-PSMA-617 therapy of prostate cancer. In case the therapeutic scheme for PSMA therapy includes PET/CT staging after every second therapy cycle, this information can be used to guide whether MTP imaging might become necessary for the subsequent therapy cycle because of large changes in tumor burden.

We recognize the limitation that our imaging protocol did not include time points after 72 h p.i. This study was based on the available imaging data at our institution—data that were acquired during the routine 3-d hospital stay for patients receiving ^177^Lu-PSMA-617 therapy. However, our ranges of collected imaging time points are comparable to those of other institutions (26*,*^29^–^31^). Further research is needed to assess the validity of our results, including time points of 96 h p.i. or later, and may lead to a different favorable time point for the STP approach for lesions due to their longer retention time (32) than was shown in our study. Our suggested imaging time point of 48 h p.i. ensured that the TIA determined with STP_prior_ was within ±20% of the TIA_ref_ for 97% of kidneys, 95% of TB_FOV_, and 86% of individual lesions (Fig. 6). However, this 48 h p.i. time point is outside the interval from 0.75 to 2.5 times the T_1/2 eff_ for about 50% of the individual lesions for cycles 1 and 2 and for 50%–60% of TB_FOV_ (Table 1). An imaging time point of 72 h p.i. may be more applicable for STP_H_ for lesions but with larger differences from TIA_ref_ for kidneys.

Patients with mCRPC may present with extensive metastases which can challenge the tracking of lesions across cycles and the calculation of ADs on an individual-lesion basis. Our analysis for individual lesions was therefore limited to 6 representative lesions per patient. Organ and lesion T_1/2 eff_ not only may depend on the individual patient but may vary widely between radiopharmaceuticals (Table 2 of Hou et al. (24) and Fig. 3 of Schuchardt et al. (33)). The applicability of different STP dosimetry approaches should therefore be carefully investigated for different organs, tumors, and radiopharmaceuticals. Future work should include organs that were outside or not entirely within the FOV of our 1-bed SPECT, as well as including all lesions per patient and expanding the analysis to other PSMA compounds. Further studies should investigate how parameters that can be acquired prior to therapy may impact T_1/2 eff_. MTP imaging may be advisable when certain parameters, such as the estimated glomerular filtration rate, are outside the reference range to precisely capture the patient-individual T_1/2 eff_. On the other hand, it can be assessed whether STP approaches are still valid but at different favorable imaging time points. Nevertheless, our results suggest that STP dosimetry is feasible for ^177^Lu-PSMA-617 therapies. We hope that these findings simplify dosimetry clinical workflows and ease the implementation of routine dosimetry in RPTs.

## CONCLUSION

The present study assessed STP image-based dosimetry for ^177^Lu-PSMA-617 therapy of prostate cancer. Use of a single SPECT/CT scan at 48 or 72 h p.i. after injection of the radiopharmaceutical led to differences from the MTP-based dosimetry that were, overall, within ±20%. Both STP_H_ and STP_prior_ were valid for ^177^Lu-PSMA-617. Since STP-based dosimetry reduces the burden for patients and the overall costs and complexity of dosimetry, it facilitates the implementation of RPT dosimetry into routine clinical practice.

## DISCLOSURE

This work was partly funded by the German Research Foundation (DFG) within the Research Training Group GRK2274 (Julia Brosch-Lenz). No other potential conflict of interest relevant to this article was reported.

## Notes

KEY POINTS **QUESTION:** Can the number of imaging time points required for dosimetry be reduced? **PERTINENT FINDINGS:** STP dosimetry is feasible using either the simplified formula by Hänscheid or a prior information approach that uses MTP imaging for the first therapy cycle with STP imaging for subsequent therapy cycles. Both methods allowed for patient-individual dosimetry for kidneys and lesions, with less than ±20% PD from MTP-based approaches. **IMPLICATIONS FOR PATIENT CARE:** Patients will benefit from personalized dosimetry and prediction of related risks and outcome.
